# Clinical Profile, Comorbidities and Therapies in Type 2 Diabetes Patients on Sitagliptin-Based Therapy in Indian Outpatient Setting

**DOI:** 10.7759/cureus.74820

**Published:** 2024-11-30

**Authors:** Khurshid A Bhat, Kiran P Singh, Hanumantha Rao Maddukuri, S N Routray, Shreya Sharma, Surendra Kumar Sharma, Kamlesh Patel, Veena Kinare, Pradip Mate, R V Lokesh Kumar

**Affiliations:** 1 Department of Endocrinology, Pearl Pharmacy, Hyderpora, IND; 2 Diabetes and Endocrinology, Fortis Medcenter, Chandigarh, IND; 3 Department of Endocrinology, Aravinda Diabetes and Endocrine Clinic, Ongole, IND; 4 Department of Cardiology, SCB Medical College and Hospital, Cuttack, IND; 5 Department of Endocrinology, Max Hospital, Dehradun, IND; 6 Department of Endocrinology, Galaxy Super Specialty Hospital, Jaipur, IND; 7 Medical Affairs, Lupin Limited, Mumbai, IND

**Keywords:** combination therapy, comorbidities, dapagliflozin, diabetes duration, sitagliptin

## Abstract

Objectives

The study was conducted to generate real-world data on prescription patterns and patient profiles for sitagliptin-based therapies in real-world outpatient settings across India.

Method

A cross-sectional, observational, multicenter, real-world prescription event monitoring (PEM) study was conducted at 1058 sites across India over six months, from 1 August 2023 to 16 January 2024. Adult type 2 diabetes patients receiving sitagliptin-based mono or combination therapies were included in the study. Primary outcomes assessed included the dosage of sitagliptin and other medications and the specific drugs combined with sitagliptin. Secondary outcomes included demographic profiles, medication history, comorbidities, and glycemic parameters.

Results

A total of 10210 patients (n=6398 males; n=3812 females) completed the study. Duration of diabetes was available for 5422 patients, of which nearly half of the patients had diabetes for 1-5 years, while around 42.3% (n=2294) of the patients had diabetes for more than five years. Comorbidities were present in 36.02% (n=3678) of the patients. Hypertension and dyslipidemia were the most common comorbidities reported in 25.3% (n=2584) and 10.42% (n=1064), respectively. Sitagliptin was administered as monotherapy or in combination with other medications. Dual combination therapy was most common, with sitagliptin 50 mg+metformin 500 mg being the predominantly prescribed regimen. Triple combination therapy with sitagliptin+dapagliflozin+metformin was also prescribed in a substantial proportion of the patients. Variations in preferred regimens were observed among patients with different comorbid conditions, with sitagliptin 100 mg+dapagliflozin 10 mg being preferred in patients with established cardiovascular disease and heart failure and sitagliptin 50 mg+metformin 500 mg in diabetic patients with hypertension, dyslipidemia, and renal disease.

Conclusion

The study findings highlighted the preferences for the use of sitagliptin and its dual as well as triple combinations as integral components of diabetes management strategies in clinical practice in Indian outpatient settings. The study also found that diabetes with ASCVD and CV risk factors influence the use of sitagliptin and dapagliflozin in combination.

## Introduction

Diabetes is a significant cause of mortality and morbidity across the globe. It has become a global epidemic in developing countries like India. Approximately three-fourths of all diabetes cases worldwide are in low and middle-income countries (LMCs) [1]. Rapid socioeconomic changes, urbanization, and unhealthy lifestyle choices mainly drive this rise in the diabetes epidemic [2].

The prevalence of type 2 diabetes mellitus (T2DM) in India has increased steadily over the past few decades by nearly 2%, reaching alarming levels [2]. According to IDF, one in seven diabetic adults worldwide resides in India. Recent estimates suggest India has over 74.9 million diabetics within the age group of 20-79 years, which has been projected to increase to 124.9 million by 2045 [1].

Patients with T2DM often present with multiple comorbidities, complicating treatment decisions and necessitating a comprehensive approach to management. Hypertension (HTN), hyperlipidemia, obesity, cardiovascular disease (CVD), and chronic kidney disease (CKD) are among the most common comorbid conditions, highlighting the complexity of diabetes management and the need for tailored therapeutic interventions [3].

Current treatment guidelines recommend a multifaceted approach toward T2DM management, including lifestyle modifications, oral antidiabetic agents, and injectable therapies. Although various guidelines propose different treatment targets, there is consensus about the individualization of the glycemic targets [4]. While newer classes of antidiabetic agents, including sodium-glucose cotransporter-2 (SGLT2) inhibitors and GLP-1 receptor agonists, have evolved as second-line therapy in current T2DM treatment algorithms, DPP-4 inhibitors, particularly sitagliptin, continue to play a significant role in T2DM management as a safe class of oral antidiabetic agent with great clinical experience of their use. DPP-4 inhibitors are also a safer choice in elderly patients [1].

Among DPP-4 inhibitors, sitagliptin is the first-in-class drug that offers several advantages, including efficacy in reducing glycated hemoglobin levels, tolerability, weight neutrality, and a low risk of hypoglycemia. Reports suggest that sitagliptin significantly reduces 0.8% mean HbA1c within the first six months of use in routine clinical practice [5]. Sitagliptin, either as monotherapy or combined with other agents such as metformin or dapagliflozin, has shown promising results in both clinical trials and real-world settings, providing clinicians with a versatile treatment option [6].

Given the widespread use of sitagliptin-based therapies for the management of type 2 diabetes in Indian patients, there is a need to evaluate prescription patterns and patient demographics in real-world clinical settings. This understanding is decisive for optimizing treatment strategies, enhancing patient outcomes, and addressing the evolving challenges diabetes presents. This cross-sectional study aims to provide insights into the utilization patterns and clinical profiles of patients receiving sitagliptin-based therapies in outpatient settings across India.

## Materials and methods

Study design and ethical considerations

This was a cross-sectional, observational, multicenter, real-world, single-arm prescription event monitoring (PEM) study conducted at 396 sites in Pan India (Appendix A). The study was conducted over six months duration, from 1st August 2023 to 16th January 2024. The study was approved by the Institutional Ethics Committee (IEC) of D Y Patil University School of Medicine, Navi Mumbai (Sitagliptin/02/LUP/2023; Dated 11th July 2023). The study is also registered under CTRI as a Clinical Trial with the registration details CTRI/2023/10/058953 (Registered on 20/10/2023). This IEC also served as the coordinating center for this multicentric study. Separate approvals were not obtained from individual local ethics committees. The study was conducted per the Indian Council of Medical Research (ICMR) and International Council on Harmonization (ICH) guidelines for Good Clinical Practice and per the principles of the Declaration of Helsinki declaration of Helsinki. Before inclusion in the study, all patients provided written consent to participate. In our study, we used electronic case report forms (eCRF) for streamlined data collection, which enabled real-time data entry, minimized errors, and facilitated remote access to data, thus enhancing efficiency and data integrity.

Patient population

The study enrolled adult patients of either gender diagnosed with type 2 diabetes mellitus and aged between 18 and 65 years. Patients visiting the outpatient clinic and prescribed sitagliptin or sitagliptin-based FDC as per the clinician's discretion were eligible to be included in the study. This also included the patients who were earlier on sitagliptin monotherapy and were switched to sitagliptin-based combination therapy. Exclusion criteria included individuals with incomplete prescription data, not willing to participate in the study, individuals with T1DM, pregnant or breastfeeding females, and patients with known severe liver or severe kidney dysfunction. As this was a PEM study, no sample size calculation was performed.

Study outcomes

The study investigated several primary and secondary outcomes to comprehensively assess the prescription patterns and patient profiles for sitagliptin-based therapies. Primary outcomes included evaluating sitagliptin therapy in terms of prescription, either as monotherapy or in combination with other drugs. Primary outcomes also included the dosage of sitagliptin and other medications and the specific drugs combined with sitagliptin. Additionally, the study evaluated the medicines utilized in combination therapies with sitagliptin, mainly focusing on two and three-drug combinations. Secondary outcomes involved evaluating the demographic profile of patients, including age, gender, height, weight, and past medication history. The secondary outcomes also included the duration of T2DM and prevalent comorbidities such as hypertension, dyslipidemia, established CVD, heart failure, mild renal disease, and active thyroid disease. Additionally, lab investigations observed were fasting plasma glucose (FPG), postprandial glucose (PPG), and glycated hemoglobin (HbA1c) levels, along with serum creatinine profile.

Statistical analysis

The statistical analysis was conducted using the Stata version 13.1 software (Stata Corp, USA). As this was a prescription-based observational study, no specific study hypothesis was tested. Descriptive statistics were used to summarize the data, and results were presented for pooled data and subgroups. For continuous variables, data were presented as the number of non-missing observations (n), mean, standard deviation (SD), median, maximum, and minimum (range). Data were presented as numbers alongside their corresponding percentage values for discrete and categorical variables.

## Results

Demography of patients, duration of disease, comorbidities, and medication history

A total of 10422 type 2 diabetes patients receiving sitagliptin-based therapies were included in the study; of these, 10210 patients (n=6398 males; n=3812 females) completed the study and were considered for data analysis.

Table 1 presents the demographic characteristics of the patients.

**Table 1:** Demography of patients (n=10210).

Parameter	Mean±SD
Age (Years)	55.27±11.43
Height (cm)	161.50±33.75
Weight (kgs)	68.26±12.72

Most patients were diagnosed with diabetes for 1 to 5 years (Table 2). Comorbidities were present in 36.02% of the studied patients. Hypertension was the most reported comorbidity in 25.31% of patients, followed by dyslipidemia in 10% of the patients. Patients with comorbidity were also reported to have established CVD, HF, mild renal disease, and active thyroid disease (Table 2).

**Table 2:** Duration of disease, comorbidities, and medication history. Abbreviations- CVD: cardiovascular disease; DPP4 inhibitors: dipeptidyl peptidase four inhibitors; GLP1RA: glucagon-like peptide 1 receptor agonist; SGLT2 inhibitors: sodium-glucose cotransporter-2 inhibitors.

	Number (%) of patients
Duration of disease (n=5422)	
<1 year	305 (5.6%)
>1-5 years	2823 (52.1%)
>5-10 years	1714 (31.6%)
>10 years	580 (10.7%)
Comorbid conditions (n=10210)	
Hypertension	2584 (25.31%)
Dyslipidemia	1064 (10.42%)
Established CVD	102 (1.00%)
Heart failure	109 (1.07%)
Renal disease	213 (2.09%)
Active thyroid disease	150 (1.47%)
Other conditions	60 (0.59%)
Medication history (n=10210)	
Metformin	2249 (22.3%)
Sulfonylurea	1250 (12.24%)
SGLT2 inhibitor	586 (5.74%)
DPP4 inhibitor	521 (5.10%)
Pioglitazone	105 (1.03%)
α-glucosidase inhibitor	96 (0.94%)
Insulin, premixed	94 (0.92%)
Insulin, basal	48 (0.47%)
Insulin, basal bolus	12 (0.12%)
GLP1RA	24 (0.24%)

Medication history was reported for 48.82% of studied patients. Most patients received metformin (22.3%) as an oral antidiabetic agent before initiating sitagliptin-based therapies, followed by sulphonylurea (12.24%).

The studied patients received sitagliptin as monotherapy, dual combination therapy, or triple combination therapy. Table 3 summarizes the prescribed sitagliptin-based treatment regimens.

**Table 3:** Prescribed sitagliptin-based monotherapy or combination therapy (N=10210). Dapa: dapagliflozin; Met: metformin; Sita: sitagliptin; XR: extended-release.

Treatment	Number (%) of patients
Sita 50 mg	475 (4.65%)
Sita 100 mg	864 (8.46%)
Sita 50 mg+Met 500 mg	3441 (33.70%)
Sita 50 mg+Met 1000 mg	315 (3.09%)
Sita 100 mg+Met 1000 mg XR	336 (3.29%)
Sita 50 mg+Dapa 5 mg	425 (4.16%)
Sita 50 mg+Dapa 10 mg	173 (1.69%)
Sita 100 mg+Dapa 5 mg	59 (0.58%)
Sita 100 mg+Dapa 10 mg	2363 (23.14%)
Sita 100 mg+Dapa 10 mg+Met 500 mg	1073 (10.51%)
Sita 100 mg+Dapa 10 mg+Met 1000 mg	686 (6.72%)

Sitagliptin monotherapy

In this study, only 13.11% (n=1339) of the patients were receiving monotherapy, with 100 mg being the most commonly prescribed dose in 8.46% (n=864) of the patients compared to the 50 mg dose prescribed in 4.65% (475) of the patients (Table 3). Among the patients receiving sitagliptin monotherapy with a known duration of diabetes, 60% of the patients had a duration of diabetes less than 5 years (Table 4).

**Table 4:** Patient distribution as per prescribed sitagliptin-based therapies and duration of diabetes (N=5422). Abbreviations- Dapa: dapagliflozin; Met: metformin; Sita: sitagliptin; XR: extended-release.

Sitagliptin-based therapies	Number (%) of patients			
	<1 year	>1-5 year	>5-10 year	>10 year
Sita 50 mg (n=191)	16 (8.38%)	98 (51.31%)	43 (22.51%)	34 (17.80%)
Sita 100 mg (n=482)	49 (10.17%)	235 (48.76%)	132 (27.39%)	66 (13.69%)
Sita 50 mg+Met 500 mg (n=1927)	110 (5.71%)	1033 (53.61%)	623 (32.33%)	161 (8.35%)
Sita 50 mg+Met 1000 mg (n=205)	13 (6.34%)	93 (45.37%)	79 (38.54%)	20 (9.76%)
Sita 100 mg+Met 1000 mg XR (n=182)	12 (6.59%)	89 (48.90%)	64 (35.16%)	17 (9.34%)
Sita 50 mg+Dapa 5 mg (n=207)	11 (5.31%)	125 (60.39%)	59 (28.50%)	12 (5.80%)
Sita 50 mg+Dapa 10 mg (n=97)	4 (4.12%)	48 (49.48%)	37 (38.14%)	8 (8.25%)
Sita 100 mg+Dapa 5 mg (n=38)	2 (5.26%)	17 (44.74%)	17 (44.74%)	2 (5.26%)
Sita 100 mg+Dapa 10 mg (n=1084)	38 (3.51%)	595 (54.89%)	307 (28.32%)	144 (13.28%)
Sita 100 mg+Dapa 10 mg+Met 500 mg (n=548)	31 (5.66%)	283 (51.64%)	185 (33.76%)	49 (8.94%)
Sita 100 mg+Dapa 10 mg+Met 1000 mg (n=340)	15 (4.41%)	140 (41.18%)	123 (36.18%)	62 (18.24%)

Mean HbA1c, FPG, and PPG values in patients receiving sitagliptin 50 mg were 7.56%, 176.58 mg/dL, and 255.47 mg/dL, respectively, while these values for sitagliptin 100 mg were 7.36 %, 169.60 mg/dL, and 230.80 mg/dL, respectively (Table 5).

**Table 5:** Prescribed sitagliptin-based therapies and laboratory parameters. Data presented as Mean±SD. Dapa: dapagliflozin; FPG: fasting plasma glucose; Met: metformin; PPG: postprandial plasma glucose; Sita: sitagliptin; XR: extended-release. Reference range for laboratory parameters-HbA1C normal range: 4.0-5.6%; HbA1C in diabetic patients: 6.5% or higher; FPG normal range: 70-100 mg/dL; FPG in diabetic patients: above 126 mg/dL; PPG normal range:<140 mg/dL; PPG in diabetic patients: 200 mg/dL or higher.

Sitagliptin-based therapies	HbA1C %	FPG mg/dL	PPG mg/dL	Creatinine mg/dL
	(n=2942)	(n=2823)	(n=1714)	(n=508)
Sita 50 mg	7.56±1.38	176.58±69.89	255.47±81.14	0.98±0.30
Sita 100 mg	7.36±121	169.60±53.70	230.80±66.78	1.15±0.31
Sita 50 mg+Met 500 mg	7.84±1.24	178.37±62.44	243.56±69.28	1.06±0.38
Sita 50 mg+Met 1000 mg	7.54±1.42	178.58±70.55	236.37±84.86	0.99±0.26
Sita 100 mg+Met 1000 mg XR	7.47±1.41	176.30±67.64	241.79±99.20	1.02±0.43
Sita 50 mg+Dapa 5 mg	8.07±1.30	150.54±53.73	239.55±83.93	0.89±0.39
Sita 50 mg+Dapa 10 mg	7.64±1.19	165.53±66.27	230.56±67.84	0.82±0.23
Sita 100 mg+Dapa 5 mg	7.58±1.10	179.46±55.87	229.75±51.61	0.96±0.09
Sita 100 mg+Dapa 10 mg	8.06±1.37	179.63±67.99	248.17±78.80	0.89±0.26
Sita 100 mg+Dapa 10 mg+Met 500 mg	7.72±1.20	168.49±81.08	234.17±87.08	0.87±0.29
Sita 100 mg+Dapa 10 mg+Met 1000 mg	8.18±1.70	151.99±73.28	216.11±101.45	0.88±0.26

In the studied population, 11.2% of the patients with hypertension were receiving sitagliptin monotherapy; among these, 11%, 10.1%, and 5.2% of patients receiving sitagliptin monotherapy presented with HF, dyslipidemia, and CVD, respectively (Table 6).

**Table 6:** Patient distribution as per prescribed sitagliptin-based therapies and comorbid conditions. ATD: active thyroid disease; CVD: cardiovascular disease; Dapa: dapagliflozin; DLD: dyslipidemia; HF: heart failure; HTN: hypertension; Met: metformin; RD: renal disease; Sita: sitagliptin; XR: extended-release.

	Number (%) of patients					
	HTN (n=2584)	DLD (n=1064)	CVD (n=102)	HF (n=109)	RD (n=213)	ATD (n=150)
Sita 50 mg	107 (4.1%)	34 (3.2%)	3 (2.9%)	4 (3.7%)	2 (0.9%)	0 (0.0%)
Sita 100 mg	184 (7.1%)	73 (6.9%)	4 (2.3%)	8 (7.3%)	6 (2.8%)	4 (2.7%)
Sita 50 mg+Met 500 mg	807 (31.2%)	337 (31.7%)	23 (22.5%)	22 (20.2%)	56 (26.3%)	41 (27.3%)
Sita 50 mg+Met 1000 mg	88 (3.4%)	30 (2.8%)	3 (2.9%)	3 (2.8%)	16 (7.5%)	14 (9.3%)
Sita 100 mg+Met 1000 mg XR	111 (4.3%)	38 (3.6%)	3 (2.9%)	0 (0.0%)	18 (8.5%)	17 (11.3%)
Sita 50 mg+Dapa 5 mg	132 (5.1%)	24 (2.3%)	3 (2.9%)	3 (2.8%)	7 (3.3%)	6 (4.0%)
Sita 50 mg+Dapa 10 mg	43 (1.7%)	29 (2.7%)	0 (0.0%)	2 (1.8%)	6 (2.8%)	4 (2.7%)
Sita 100 mg+Dapa 5 mg	17 (0.7%)	4 (0.4%)	1 (1.0%)	2 (1.8%)	0 (0.0%)	0 (0.0%)
Sita 100 mg+Dapa 10 mg	602 (23.3%)	287 (27.0%)	41 (40.2%)	44 (40.4%)	50 (23.5%)	24 (16.0%)
Sita 100 mg+Dapa 10 mg + Met 500 mg	244 (9.4%)	107 (10.1%)	15 (14.7%)	1 (10.1%)	24 (11.3%)	16 (10.7%)
Sita 100 mg+Dapa 10 mg + Met 1000 mg	216 (8.4%)	72 (6.8%)	8 (7.8%)	4 (3.7%)	27 (12.7%)	22 (14.7%)

Sitagliptin dual combination therapies

Most of the studied patients were receiving dual combination therapy, with sitagliptin 50 mg+metformin 500 mg being the most commonly prescribed dual combination in 33.70% of the patients, followed by sitagliptin 100 mg+dapagliflozin 10 mg in 23.14% of the patients. Other dual combination therapies prescribed were sitagliptin 50 mg+metformin 1000 mg, sitagliptin 100 mg + metformin 1000 mg extended-release (XR), sitagliptin 50 mg+dapagliflozin 5 mg, sitagliptin 50 mg+dapagliflozin 10 mg and sitagliptin 100 mg+dapagliflozin 5 (Table 3). Among the patients receiving sitagliptin 50 mg+metformin 500 mg with known status of duration of diabetes, 53.61% of patients had diabetes for 1 to 5 years, while 40.68% had diabetes for more than 5 years (Table 4). Among the patients receiving sitagliptin 100 mg+dapagliflozin 10 mg, the duration of diabetes was 1 to 5 years in 54.89% of patients and more than 5 years in 41.60% of the patients (Table 4). Evaluation of laboratory parameters showed that in patients receiving sitagliptin 50 mg+metformin 500 mg, the values of HbA1c, FPG, and PPG were 7.84%, 178.37 mg/dL and 243.56 mg/dL, respectively (Table 5). In patients receiving sitagliptin 100 mg+dapagliflozin 10 mg, these values were 8.06%, 179.63 mg/dL, and 248.17 mg/dL, respectively (Table 6). Hypertension and dyslipidemia were the most common comorbidities in patients receiving sitagliptin 50 mg+metformin 500 mg, with prevalence of 31.2% and 31.7% of the total population, respectively. CVD was present in 22.5% of patients, HF in 20.2% of patients, and renal disease in 26.3% of patients receiving sitagliptin 50 mg metformin 500 mg (Table 6). Higher prevalence of CVD and HF were reported in patients receiving sitagliptin 100 mg+dapagliflozin 10 mg. Around 40.2% of CVD patients and 40.4% of HF patients were present in the sitagliptin 100 mg+dapagliflozin 10 mg group. Also, 23.3% of hypertensive patients, 27.0% of patients, and 23.5% of renal disease patients were in this group (Table 6).

Sitagliptin triple combination therapies

Triple combination therapy was prescribed in overall 1763 of the (17.23%) patients, where 1076 (10.51%) were on sitagliptin 100 mg + dapagliflozin 10 mg+metformin 500 mg and 686 (6.72%) were on sitagliptin 100 mg+dapagliflozin 10 mg+metformin 1000 mg. Most of the triple therapy user patients (47.15%) had diabetes for 1 to 5 years, 46.71% had diabetes for more than 5 years, while only 5.1% of the patients receiving triple combination therapy had a duration of diabetes <1 year (Table 4). Mean HbA1c, FPG, and PPG values in patients on sitagliptin 100 mg+dapagliflozin 10 mg+metformin 500 mg were 7.72, 151 mg/dL, and 216 mg/dL, respectively (Table 5). For patients receiving sitagliptin 100 mg+dapagliflozin 10 mg+metformin 1000 mg triple combination, these values were 8.19%, 168.49 mg/dL, and 234.17 mg/dL, respectively (Table 5). Among the patients with comorbidities, 17.8% of patients with hypertension, 16.9% with dyslipidemia, 22.5% with CVD, 13.8% with HF, and 24% of the patients with renal disease were receiving triple combination therapy (Table 6).

Differences in the preferred sitagliptin-based regimens were seen when the subgroup of patients with different comorbid conditions was considered. Sitagliptin 50 mg+metformin 500 mg was the most preferred combination in patients with hypertension, dyslipidemia, and renal disease, and sitagliptin 100 mg+dapagliflozin 10 mg was the second most commonly prescribed combination, as seen in overall population data. However, sitagliptin 100 mg+dapagliflozin 10 mg was the most frequently prescribed combination in nearly 40% of the patients with established CVD and those with HF. The second most prescribed combination of these two subgroups was sitagliptin 50 mg+metformin 500 mg (Table 6).

The results showed that HbA1C levels were significantly lower (p<0.0001) in the sitagliptin monotherapy group compared to other dual and triple drug combinations studied. The HbA1C levels in the sitagliptin+metformin groups were also lower than in the sitagliptin+dapagliflozin groups (p<0.0001). While the HbA1C levels in sitagliptin+metformin+dapagliflozin groups were not significantly different from both dual therapy groups (Table 5). There was no significant difference in FPG and PPG levels between the group of patients receiving either therapy. However, creatinine levels were significantly different. Creatinine levels were significantly higher (p<0.0001) in monotherapy groups compared to sitagliptin+dapagliflozin dual and triple therapy groups. Also, the creatinine levels in the sitagliptin+metformin group were significantly higher (p<0.0001) than in these two patient groups. There was no significant difference between creatinine levels of patients in sitagliptin+metformin dual therapy and triple therapy groups.

## Discussion

The findings of this study shed light on the current landscape of diabetes management in India, with a particular focus on the utilization patterns and patient demographics associated with sitagliptin-based therapies.

With nearly two decades of clinical experience, dipeptidyl peptidase-4 (DPP-4) inhibitors are a well-established class of oral hypoglycemic agents, offering clinicians a safe and effective therapeutic option in the multifaceted management of T2DM [1,7]. By inhibiting DPP-4 enzyme activity, these agents potentiate the actions of incretin hormones, namely glucagon-like peptide-1 (GLP-1) and gastric inhibitory polypeptide (GIP), addressing key pathophysiological aspects of diabetes [8]. Despite the emergence of newer agents like sodium-glucose cotransporter-2 (SGLT2) inhibitors and GLP-1 receptor agonists, DPP-4 inhibitors have a significant role in diabetes management [1]. Their favorable safety profile, anti-inflammatory properties, and minimal risk of hypoglycemia or weight gain make them a valuable option, particularly in elderly patients and those with CKD, where other agents may have limitations [7].

Sitagliptin is the first in the class of DPP-4 inhibitors, and it received approval in 2006 for the management of T2DM. In clinical studies, sitagliptin has demonstrated a reduction of up to 0.7% in HbA1c, which has also been consistently reported in real-world clinical settings with a substantial reduction of mean HbA1c up to 0.8% [5]. Sitagliptin helps achieve target HbA1c levels and reductions in fasting and postprandial glucose levels. Clinical trials have also reported a favorable safety profile of sitagliptin with minimal risk of hypoglycemia depending on background therapy and a neutral impact on body weight [5,6]. The STREAM study, which evaluated sitagliptin in older adults with moderately controlled T2DM, further reinforced sitagliptin's clinical efficacy and safety for diverse patient populations, including older adults with type 2 diabetes [9]. Clinical trials have highlighted the efficacy of sitagliptin across various treatment regimens, including monotherapy, initial combination therapy, typically with sitagliptin-metformin FDC, or adjunct therapy to metformin or other antihyperglycemic drugs, including SGLT-2 inhibitors as well as insulin therapy [5,6]. Our study identified that many patients received sitagliptin in combination with metformin and dapagliflozin. Triple FDC, composed of metformin, sitagliptin, and dapagliflozin, has also been prescribed in a substantial proportion of T2DM patients receiving sitagliptin-based therapies. In this study, fewer patients were receiving sitagliptin monotherapy.

The therapeutic efficacy of sitagliptin and metformin combination therapy in T2DM has been extensively evaluated in clinical trials. This combination demonstrated superior glycemic improvements compared to metformin alone in antihyperglycemic treatment-naive patients [10]. The sitagliptin and metformin as initial combination therapy demonstrated significant reductions in HbA1c and fasting plasma glucose levels and a higher proportion of patients achieving target HbA1c levels of <7% [10]. The COSMIC study, a real-world follow-up study, demonstrated the four-year durability of initial combination therapy with sitagliptin and metformin in patients with type 2 diabetes in clinical practice [11]. A randomized, double-blinded study also showed that sitagliptin+metformin as an initial treatment leads to more significant improvements in glycemic control and body weight changes, with a lower incidence of hypoglycemia than glimepiride [12]. Furthermore, the addition of sitagliptin to ongoing metformin therapy in patients with uncontrolled diabetes has demonstrated sustained benefits over placebo plus metformin [13]. Sitagliptin, as an add-on to metformin, also has shown noninferiority against sulfonylurea, glipizide [14], or glimepiride [15] added onto metformin in patients with uncontrolled diabetes. These findings support the efficacy and sustainability of sitagliptin/metformin combination therapy as an effective treatment option in managing T2DM, whether as initial therapy or as an add-on to ongoing metformin treatment regimens [16]. In our study, 40% of the patients were on sitagliptin+metformin combination therapy, with sitagliptin 50 mg+metformin 500 mg being the most commonly prescribed dual combination in nearly 34%.

The integration of sitagliptin with SGLT2 inhibitors, such as dapagliflozin, in fixed-dose combinations has shown promising outcomes in patients with uncontrolled T2DM. The combination therapy of dapagliflozin and sitagliptin demonstrates promising efficacy and safety in managing T2DM, as revealed by clinical trials [17,18]. In a multicenter randomized trial, dapagliflozin as an add-on to sitagliptin, with or without metformin, significantly reduced mean HbA1c levels by -0.5% and body weight compared to placebo over 24 weeks, with sustained benefits observed through 48 weeks [17]. Supporting these findings, the Real DAPSI trial, a real-world retrospective study conducted in India, documented the effectiveness and safety of the dapagliflozin-sitagliptin fixed-dose combination, demonstrating significant reductions in mean HbA1c by 1.7% and significant reductions in fasting, and postprandial blood glucose levels without serious adverse events reported over 12 weeks [18]. Together, these results emphasize the clinical utility of dapagliflozin-sitagliptin combination therapy in optimizing glycemic control in T2DM patients, providing valuable tools for clinicians in tailoring treatment strategies and improving patient outcomes. In our study, nearly 30% of the patients received sitagliptin+dapagliflozin combination therapy, whereas almost 23% received sitagliptin 100 mg+dapagliflozin 10 mg combination. This implies that sitagliptin+dapagliflozin is the second most commonly prescribed sitagliptin-based dual combination in real-world clinical settings.

As mentioned above, dapagliflozin added to sitagliptin with or without metformin is effective and safe in managing T2DM, highlighting the benefits of this triple combination [17]. Another two major clinical trials supported the clinical utilization of this triple combination for T2DM. A phase 3 study compared FDC of sitagliptin+metformin+dapagliflozin with dual combinations, sitagliptin+metformin, and dapagliflozin+metformin, in patients inadequately controlled with metformin. Results of this study showed that the triple combination was significantly more effective in reducing HbA1c levels than the dual combinations, with superior reductions in postprandial and fasting blood glucose levels and a higher proportion of patients achieving HbA1c<7.0%. Safety profiles were similar across groups [19]. The second trial, the MESIDA trial, which was a randomized, double-blind study, compared a triple-drug FDC of sitagliptin+metformin+dapagliflozin with a two-drug combination of sitagliptin+metformin in Indian patients with T2DM. In this trial, triple combination demonstrated superior reductions in HbA1c, fasting plasma glucose, and postprandial glucose compared to dual combination, with comparable safety profiles [20]. Current clinical evidence highlights that sitagliptin-based triple combination with metformin and dapagliflozin represents a promising approach for managing T2DM, offering enhanced glycemic control and comparable safety profiles compared to dual combinations. In our study, around 17% of the patients received sitagliptin+metformin+dapagliflozin triple combination therapy, where sitagliptin 100 mg+dapagliflozin 10 mg+metformin 500 mg was the most prescribed triple combination in 10.51% of the patients.

In this study, we also correlated the prescription pattern of sitagliptin-based therapies with patient characteristics. There was heterogeneity in the clinical presentation of diabetes in the studied patients. In our study, most of the patients receiving sitagliptin-based therapies had a longer duration of diabetes. More than half of the patients with reported study duration had diabetes for 1 to 5 years, while around 40% of the patients had diabetes for more than five years. When we studied the prescription pattern of sitagliptin-based therapy with respect to the duration of diabetes, it showed that most of the patients on triple combination therapy had a longer duration of diabetes compared to those receiving monotherapy.

Patients with T2DM frequently present with multiple comorbidities, which also impacts the selection of strategies for diabetes treatment. These comorbidities are more prevalent in older age groups and are often higher in men than women. Hypertension, overweight/obesity, hyperlipidemia, chronic kidney disease (CKD), and cardiovascular disease are among the most common conditions observed in patients with T2DM, with hypertension being the most prevalent condition reported in nearly 80% of diabetic patients [3,21]. In this study also, comorbidities were present in a significant proportion of the patients, where hypertension and dyslipidemia were the most common co-existing conditions. Other commonly reported comorbidities were c ardiovascular disease (CVD), heart failure (HF), mild renal disease, and active thyroid disease. When the prescription pattern of sitagliptin-based therapy was studied with respect to present comorbidities, differences were observed in the selection of dual combination therapies. In diabetic patients with the presence of comorbidities, including hypertension, dyslipidemia, and renal disease, the preferences were unchanged, with sitagliptin 50 mg+metformin 500 mg being the most preferred regimen. However, in patients with established CVD and those with HF, sitagliptin 100 mg+dapagliflozin 10 mg was the most preferred therapy.

Sitagliptin has multiple pleiotropic effects beyond glycemic control. When sitagliptin was evaluated in clinical trials for its effect on blood pressure, it was found that sitagliptin slightly but significantly reduced BP from 6 months after initiation of therapy [22]. In clinical trials, sitagliptin has been shown to be beneficial in diabetic patients with dyslipidemia. The GLORIA trial showed that sitagliptin significantly reduces fasting triglycerides (TG), low-density lipoprotein cholesterol (LDL-C), and non-high-density lipoprotein cholesterol (non-HDL-C) levels. Additionally, sitagliptin also significantly decreases total cholesterol (TC) levels [23]. All this evidence points towards a preference for sitagliptin therapy in diabetic patients with hypertension, dyslipidemia, and renal disease as comorbidities, as seen in our study. Sitagliptin is also well known for its reno-protective action. In diabetic patients, sitagliptin reduces urinary albumin excretion without affecting the glomerular filtration rate [24]. Preference for sitagliptin+dapagliflozin combination therapy echoes the cardiovascular benefits of dapagliflozin observed in clinical trials. In the DAPA-HF trial, dapagliflozin demonstrated a reduced risk of cardiovascular death, heart failure (HF) hospitalizations, and all-cause mortality in a wide range of HF patients [25]. Dapagliflozin has also proved to lower the rate of cardiovascular death or hospitalization for heart failure in T2DM patients at risk for atherosclerotic cardiovascular disease [26]. This evidence makes sitagliptin+dapagliflozin a rational combination to be prescribed in diabetic patients with comorbid CVD and HF, as seen in this study.

Medication history taking in this study showed that nearly half (48.83%) of the patients were on other antidiabetic drugs before initiation of the sitagliptin-based therapies, while the other half didn't receive any antidiabetic-drug, prior to initiation of the sitagliptin-based therapies. This shows that sitagliptin-based therapies are equally prescribed as first-line therapy and as second-line therapy/add-on to other antidiabetic drugs. Sitagliptin is effective in both clinical settings, as seen in multiple clinical trials.

The observed prescription patterns in this study revealed the versatility of sitagliptin-based therapies in managing diabetes. This reflects the recognition of sitagliptin's efficacy, tolerability, and safety profile among healthcare providers, aligning with current treatment guidelines advocating for individualized approaches to diabetes management.

This study has some limitations. As an observational, cross-sectional analysis, it cannot establish causality between sitagliptin-based therapies and clinical outcomes, limiting insights into long-term efficacy and safety. The study relied on real-world prescription patterns from outpatient clinics across India, which could introduce selection bias and may not fully represent broader or inpatient populations. These factors suggest areas for further research to comprehensively assess sitagliptin's role in diabetes management.

## Conclusions

Overall, the study provided a comprehensive evaluation of the real-world prescription patterns of sitagliptin-based therapy and the profile of patients with type 2 diabetes receiving them in outpatient settings across India. The study findings highlighted the widespread use of sitagliptin and its dual and triple combinations as integral components of diabetes management strategies in clinical practice. The study also found that diabetes with atherosclerotic cardiovascular disease and cardio vascular risk factors influence the use of sitagliptin and dapagliflozin in combination.

## Appendices

Appendix A: The following is the list of the 396 centres utilized in the study.

**Table 7:** Centre list

Center Name	City	State
Shri Sant Achyut Maharaj Hospital	Amravati	Maharashtra
Ramsudha Medical	Guwahati	Assam
Amey Hospital	Kolhapur	Maharashtra
Patil Clinic	Kolhapur	Maharashtra
Aarush Clinic	Coimbatore	Tamil Nadu
Santhi Social Service Hospital	Coimbatore	Tamil Nadu
Swad Diabetes Care Centre	Sangli	Maharashtra
Star Hospital	Srikakulam	Andhra Pradesh
Jain Hospital	Jalgaon	Maharashtra
Dr Sai Swaroop	Hindupur	Andhra Pradesh
Medicover Hospital	Kakinada	Andhra Pradesh
Vaishnavi Hospital	Guntur	Andhra Pradesh
Diabetic Clinic		Chattisgarh
Aapyaa Hospital	Bhimavaram	Andhra Pradesh
Unitaas Hospital	Chennai	Tamil Nadu
Sree Ramchandra Nursing Home	Rajahmundry	Andhra Pradesh
Sandipan Sarkar	Burdwan	West Bengal
Maddukuri Hanumantharao	Ongole	Andhra Pradesh
A. V Bhavesh	Kakinada	Andhra Pradesh
Hari Clinic	Madurai	Tamil Nadu
Padmaja Clinic	Nuzvid	Andhra Pradesh
Spandana Hospital	Bhimavaram	Andhra Pradesh
P Ramnaresh	Palasa Kasibugga	Andhra Pradesh
Amrutha Laxmi Multispeciality Hospital	Nizamabad	Telangana
Sks Hospital	Coimbatore	Tamil Nadu
Kuna Mohan Babu	Palasa Kasibugga	Andhra Pradesh
Sooriya Heart Care	Pondicherry	Tamil Nadu
Med Fit Clinic	Mangalore	Karnataka
Vinayaka Speciality Clinic	Davangere	Karnataka
J V Hospital	Chennai	Tamil Nadu
Anugraham Clinic	Madurai	Tamil Nadu
Kmc Hospital	Mangalore	Karnataka
Dr Patil Clinic	Davangere	Karnataka
Shrawasti Hospital	Kolhapur	Maharashtra
Vijaya Hospital	Narasapur	Andhra Pradesh
Sai Bramanambika	Razole	Andhra Pradesh
Jayabharath Hospital	Nellore	Andhra Pradesh
Fortis Hospital	Chennai	Tamil Nadu
Ngj Hospital	Madurai	Tamil Nadu
Kgmu	Lucknow	Uttar Pradesh
Muktai Hospital	Nashik	Maharashtra
Sajeevani Hospital Kollakadavu682004	Trivandrum	Kerala
Chaitanya Hospital	Tirupati	Andhra Pradesh
Medanta Hospital	Lucknow	Uttar Pradesh
Holycare	Guwahati	Assam
Subha Rao Clinic	Kavali	Andhra Pradesh
Sir Sundarlal Hospital	Varanasi	Uttar Pradesh
Center For Dibeties	Lucknow	Uttar Pradesh
Dumpala Vidyasagar	Srikakulam	Andhra Pradesh
Vipin Gupta Clinic	Aligarh	Uttar Pradesh
Sainath Clinic	Kakinada	Andhra Pradesh
Nidaan Healthcare	Guwahati	Assam
Ramkrishna Hospital	Coimbatore	Tamil Nadu
Gayathri Hospital	Nizamabad	Telangana
Kms Clinic	Trichy	Tamil Nadu
Maa Mahamaya Medical	Guwahati	Assam
Apollo Clinic	Bangalore	Karnataka
Hebbal Heart Care Centre	Gulbarga	Karnataka
Rk Multispeciality Hospital Julywada	Warangal	Telangana
Krs Multispeciality Hospital	Shimoga	Karnataka
Anjan Saikia	Dibrugarh	Assam
Sadhan Biswas	Burdwan	West Bengal
Sri Ponni Clinic	Coimbatore	Tamil Nadu
Diabetes Thyroid &Obesity Clinic		Chattisgarh
Agnibha Maiti	Kolkata	West Bengal
Soumendra Nath Ghosh	Kolkata	West Bengal
Debtanu Banerjee	Kolkata	West Bengal
Goutam Banerjee	Burdwan	West Bengal
Ranadeb Roy	Kolkata	West Bengal
Partha Sarathi Sengupta	Kolkata	West Bengal
Shyamantak Chakraborty	Kolkata	West Bengal
Metro Hospital	Guwahati	Assam
Puja Mahato	Kolkata	West Bengal
Nivedita Sen	Kolkata	West Bengal
Tulasigireesh Hospital	Bijapur	Karnataka
Ali’s Plus Health	Pune	Maharashtra
Goyal Clinic	Agra	Uttar Pradesh
Shriram Clinic	Pune	Maharashtra
Sougata Sarkar	Burdwan	West Bengal
Shibaditya Chakraborty	Burdwan	West Bengal
Bhise Hospital	Pune	Maharashtra
Manzoor N	Perinthalmanna	Kerala
Gnrc Good Health	Guwahati	Assam
Sanjeevuni Clinic	Hyderabad	Telangana
Bala Ji Hospital	Kangra	Himachal Pradesh
Sarji Hospital	Shimoga	Karnataka
Himadri Das	Kolkata	West Bengal
Radhe Shyam Agrawal	Kolkata	West Bengal
Anashuyak Roy	Kolkata	West Bengal
Hrishikesh Majumder	Kolkata	West Bengal
Rajlakshmi Polyclinic	Kolkata	West Bengal
Satya Narayan Bajaj	Patna	Bihar
Victor Saha	Kolkata	West Bengal
Kohli Clinic	New Delhi	Delhi
Nayyar Hospital	Amritsar	Punjab
Garima Hospital	Meerut	Uttar Pradesh
Kohetoor Multispeciality Hospital	Meerut	Uttar Pradesh
Baidyabati	Kolkata	West Bengal
Amitava Ghosh	Burdwan	West Bengal
Sougata Ghosh	Burdwan	West Bengal
Ayursundra Hospital	Guwahati	Assam
Arpit Kumar Saha	Burdwan	West Bengal
Utsa Basu	Kolkata	West Bengal
Abhisek Kolay	Burdwan	West Bengal
Anirudda Saha	Kolkata	West Bengal
Dilip Kumar	Patna	Bihar
Suraj Hanumant Chavan	Ranchi	Jharkhand
Arindam Chatterjee	Kolkata	West Bengal
Sai Hospital	Meerut	Uttar Pradesh
Samim Ahmad	Patna	Bihar
Raj Clinic	Lucknow	Uttar Pradesh
Beant Singh Mann	Haryana	Himachal Pradesh
Krashana Hospital	Agra	Uttar Pradesh
J R Diabetes Center	Chennai	Tamil Nadu
Raveendran Clinic	Chennai	Tamil Nadu
Prashant Kumar	Patna	Bihar
Chaitanya Hospital And Nursing Home	Pune	Maharashtra
Nagarmal Goyal	Sikar	Rajasthan
Anandrushiji Hospital	Ahmednagar	Maharashtra
Jj Diabetes And Obecity Clinic	Ahmednagar	Maharashtra
Giridhar Clinic	Pune	Maharashtra
Joshi Clinic	Pune	Maharashtra
Mahesh Hakke	Mumbai	Maharashtra
Ahana Clinic	Lucknow	Uttar Pradesh
Sri Raghavendra Clinic	Davangere	Karnataka
Raj Hospital Ranchi	Ranchi	Jharkhand
City Hospital	Meerut	Uttar Pradesh
Mathi Clinic	Madurai	Tamil Nadu
S A Pathy Clinic	Madurai	Tamil Nadu
Dr Mukesh Mehrotra Clinic	Jabalpur	Madhya Pradesh
Gandhi Clinic		Madhya Pradesh
Mediva Hospital	Jaipur	Rajasthan
Sion Hospital	Mumbai	Maharashtra
Bala Diabetic Care	Chennai	Tamil Nadu
Apollo Clinic	Hyderabad	Telangana
Anand Daignostic Center	Bangalore	Karnataka
Krishnamoorthy Clinic	Madurai	Tamil Nadu
Syed Maqsood Husain	Bhopal	Madhya Pradesh
Medicover	Hyderabad	Telangana
P Das	Kolkata	West Bengal
Sarvadnya Hospital	Pune	Maharashtra
Dr. Amitava Singh	Mathura	Uttar Pradesh
Swastik Health Care	Mathura	Uttar Pradesh
Siddhivinayak Clinic	Pune	Maharashtra
Goroshi Clinic	Belgaum	Karnataka
Sagnik Mukhopadhyay	Kolkata	West Bengal
Supriyo Saha	Kolkata	West Bengal
Chanchal Das	Kolkata	West Bengal
R Chowdhury	Kolkata	West Bengal
Soumyadeep Ghosh	Kolkata	West Bengal
Umeed Singh	Sikar	Rajasthan
Chaitanya Nursing Home	Pune	Maharashtra
Dr Sumit Chatterjee	Kolkata	West Bengal
Garg Clinic	Lucknow	Uttar Pradesh
Dr K I Rasul	Muzaffarpur	Bihar
Ram Shindhe	Mumbai	Maharashtra
Saimauli Hospital	Ahmednagar	Maharashtra
Jayadeva Hospital	Mysore	Karnataka
Dr. N.T Shaha Multi-Speciality Clinic	Pune	Maharashtra
Anil Joshi	Jaipur	Rajasthan
Wellness Clinic	Pune	Maharashtra
Shriyash Clinic	Pune	Maharashtra
Dr Mohan Hospital	Thanjavur	Tamil Nadu
Sree Vidya Clinic	Chennai	Tamil Nadu
Kulkarni Hospital	Latur	Maharashtra
Asharfi Hospital	Varanasi	Uttar Pradesh
Kims Hospital Kondapur	Hyderabad	Telangana
Deep Ghoswami	Kolkata	West Bengal
Navjivan Hospital	Aurangabad	Maharashtra
Dombale Hospital	Satara	Maharashtra
Shivnandan Hospital	Pune	Maharashtra
Sami Hospital	Aurangabad	Maharashtra
Dr. Taiyab Clinic	Bhopal	Madhya Pradesh
Amrith Hospital	Chennai	Tamil Nadu
Dr P K Jena	Cuttack	Odisha
Sum Hospital	Bhubaneshwar	Odisha
Gajraj Hospital	Delhi	Haryana
Rajan Clinic	Ranchi	Jharkhand
Om Prakash Saha	Patna	Bihar
Kims	Bhubaneshwar	Odisha
Yousufs Clinic	Hyderabad	Telangana
Dr Nigamananda Tripathy	Cuttack	Odisha
Dr Durga Prasad Das	Cuttack	Odisha
Apollo Clinic	Pune	Maharashtra
Manjeet Singh Trehan	Chandigarh	Himachal Pradesh
Kallaem Bhumareddy Hospital	Nizamabad	Telangana
Dr Sitakanta Panda	Cuttack	Odisha
Sandeep Mahla	Sikar	Rajasthan
Namit Mathur	Jodhpur	Rajasthan
Dr Arun Dalmia	Cuttack	Odisha
Jeevan Anmol Hospital	Delhi	Delhi
Medi Centre	Ranchi	Jharkhand
Endocrine Super Speciality Clinic	Davangere	Karnataka
Medibone Hospital		Chattisgarh
Nair Hospital	Mumbai	Maharashtra
Dr Abdhut Pradhan	Cuttack	Odisha
Shri Ram Shakti Clinic	Lucknow	Uttar Pradesh
Dr L Ravikumar	Cuttack	Odisha
Nri Hospital	Mangalagiri	Andhra Pradesh
Shri Jagannath Clinic	Bhubaneshwar	Odisha
Perwez Aziz	Muzaffarpur	Bihar
Anitha Hospital	Madurai	Tamil Nadu
Baheti Hospital	Nanded	Maharashtra
Ramesh Hospital	Vijayawada	Andhra Pradesh
Saptagiri Hospital	Bangalore	Karnataka
Clinic	New Delhi	Jammu And Kashmir
Dr Deepankar Shivam	Meerut	Uttar Pradesh
Sut Hospital	Trivandrum	Kerala
Vijay Clinic	Coimbatore	Tamil Nadu
Debashish Mondal	Kolkata	West Bengal
Sri Saravana Clinic	Coimbatore	Tamil Nadu
Kumaran Hospital	Coimbatore	Tamil Nadu
Pankaj Seth	Punjab	Himachal Pradesh
Dua Medical Centre	Ludhiana	Punjab
Ajas Jamal	Trivandrum	Kerala
Mrityunjay Heart Care Center	Meerut	Uttar Pradesh
Sanchita Majumdar	Dibrugarh	Assam
Anirban Dutta	Kolkata	West Bengal
Manish Dhadke	Mumbai	Maharashtra
Manoj Kumar Badam	Nizamabad	Telangana
Kvr Hospital	Meerut	-
Arun Medical Center	Pondicherry	Tamil Nadu
Sai Sivam Clinic	Coimbatore	Tamil Nadu
Pankaj Nawal Bihari	Patna	Bihar
Aanandam Hospital	Nagpur	Maharashtra
A R Hospital	Madurai	Tamil Nadu
Satyajit Parhi	Bhubaneshwar	Odisha
Ankan Jha	Mumbai	Maharashtra
Sathya Specality	Bangalore	Karnataka
Suchetana Goswami	Siliguri	West Bengal
Clinic	New Delhi	Delhi
Satish Kumar Prasad	Ranchi	Jharkhand
Devendra Patel	Mumbai	Maharashtra
Pallavi Medical	Guwahati	Assam
Gandhi Clinic	Satara	Maharashtra
Dr Sudipta Rout	Cuttack	Odisha
Living Well Health Clinic	Faridabad	Haryana
Dibakar Sinha	Siliguri	West Bengal
Suvidha Hospital	Chandur Bazar	Maharashtra
Happy Clinic	Jabalpur	Madhya Pradesh
Kalyan Kumar Das	Siliguri	West Bengal
Sakshi Gangneja	Jodhpur	Rajasthan
Saurav Dey	Kolkata	West Bengal
Dr Satyams Clinic	Warangal	Telangana
Rajesh Ranjan	Patna	Bihar
Subrata Chakrabarti	Kolkata	West Bengal
Diptak Bhowmick	Kolkata	West Bengal
Pradeep Kumar	Patna	Bihar
Soumyadeep Ghosh	Burdwan	West Bengal
Sayan Das	Burdwan	West Bengal
Narayani Hospital	Chennai	Tamil Nadu
Navin R	Kolathur	Tamil Nadu
K Sandhyarani	Karimnagar	Telangana
Tade Clinic	Pune	Maharashtra
Diabetes Care	Guwahati	Assam
Guruvinder Singh	Punjab	Himachal Pradesh
Anudeep Heart Center	Varanasi	Uttar Pradesh
Anil Samaria	Ajmer	Rajasthan
Praveen Rao Endo Care Hospital	Warangal	Telangana
City Super Speciality Hospital	Kangra	Himachal Pradesh
Munot Heart Care & Research Centre	Pune	Maharashtra
Nilesh Bajaranglal Chandak	Amravati	Maharashtra
Sandeep Kumar	Hanumangarh	Rajasthan
Rajesh Kumar Pandey	Jaipur	Rajasthan
Mohit Goyal	Punjab	Himachal Pradesh
Mahesh Narendra Jain	Udaipur	Rajasthan
Mahesh Rath	Bhubaneshwar	Odisha
Madina Clinic	Hyderabad	Telangana
Dr U K Pandey Clinic	Varanasi	Uttar Pradesh
Dr. Madhu Gupta	Mumbai	Maharashtra
Lmh Bardi Hospital	Nagpur	Madhya Pradesh
Moiz Hussain	Bhopal	Madhya Pradesh
Patel Chirag Prahladbhai	Ahmedabad	Gujarat
Rahul Khanna	Haryana	Haryana
Vighnesh Rane	Mumbai	Maharashtra
Swastik Hospital	Jaipur	Rajasthan
Synapse Neuro Center	Bangalore	Karnataka
Ravi Kant Soni	Sikar	Rajasthan
Shiv Prakash Sharma	Jaipur	Rajasthan
Ehcc Hospital	Jaipur	Rajasthan
Vansh Clinic	Hamirpur	Himachal Pradesh
Nitika Sharma	Hamirpur	Himachal Pradesh
Ravi	Mumbai	Maharashtra
Swati Srivastav	Jaipur	Rajasthan
Wellness Hospital	Panvel	Maharashtra
Katharia Hospital	Surat	Gujarat
Chirayu Praful Vyas	Ahmedabad	Gujarat
Dr Abhishek Kumar	Muzaffarpur	Bihar
Vinayak Hospital	Meerut	-
Navdeep Singh Suri	Punjab	Himachal Pradesh
Care Hospital	Hyderabad	Telangana
Global Sunshine Hospital	Baroda	Gujarat
Raj Lal Bhagat	Punjab	Himachal Pradesh
Awanti Hospital		Chattisgarh
Vimal Bagga	Mohali	Punjab
Raylen Hospital	Ahmedabad	Gujarat
Dr. Gaurav Gupta	Firozabad	Uttar Pradesh
Anand Hospital	Kangra	Himachal Pradesh
Vipul P Chavda	Ahmedabad	Gujarat
Dhruvi Hasnani	Ahmedabad	Gujarat
Nilkanth Hospital	Pune	Maharashtra
Endocrine And Diabetes Clinic	Faridabad	Haryana
Navjivan Hospital	Ahmedabad	Gujarat
Om Medical Center	Lucknow	Uttar Pradesh
Rathod Jignesh Raysinh	Baroda	Gujarat
Vivek Ved Prakash Arya	Ahmedabad	Gujarat
Deccan Hospital	Belgaum	Karnataka
Bhirud Clinic	Panvel	Maharashtra
Kanva Hospital	Bangalore	Karnataka
Pranav Dibtic Center	Bangalore	Karnataka
Berry Clinic	Ludhiana	Punjab
Mg Hospital	Bhilwara	Rajasthan
Yashoda Hospital	Hyderabad	Telangana
Rathod Clinic	Degana	Rajasthan
Dr Rakesh Kumar	Muzaffarpur	Bihar
Avighna Clinic	Ulhasnagar	Maharashtra
Laksh Hospital	Bhandara	Maharashtra
Kriyansh Clinic	Kalyan	Maharashtra
Al-Hyat Hospital	Nizamabad	Telangana
Family Wellness Centre	Meerut	Uttarakhand
Swasthya Hospital	Surat	Gujarat
Nanki Hospital	Meerut	-
Jionath Hospital	Meerut	-
Hormocare	Nashik	Maharashtra
Borana Hospital	Udaipur	Rajasthan
Civic Heart Clinic	Ahmedabad	Gujarat
Ved Nursing Home	Ahmedabad	Gujarat
Divya Clinic	Chennai	Tamil Nadu
Satguru Hospital Mahuya	Bhavnagar	Gujarat
Jayesh Clinic	Varanasi	Uttar Pradesh
Vishwani Clinic	Agra	Uttar Pradesh
Goel Clinic	Meerut	-
Nidan Clinic	Noida	Uttar Pradesh
Kavya Diabetes Clinic	Faridabad	Haryana
Tippadareddy Siva Sekhara	Atmakur Sri Potti Sriramulu	Andhra Pradesh
Jitendra Khichar	Sikar	Rajasthan
Dr.Vinitkumar Shah	Ahmedabad	Gujarat
Praveen Kumar Bass	Bareilly	Uttar Pradesh
Kiran Hospital	Surat	Gujarat
Jagdish Bhayalal Patel	Bhavnagar	Gujarat
Hormones And Diabetese Care	Varanasi	Uttar Pradesh
Sunrise Hospital	Nashik	Maharashtra
Eqbal Ahmed	Kolkata	West Bengal
Sougat Sarkar	Burdwan	West Bengal
Shanti Multispeciality Hospital	Ahmedabad	Gujarat
Krishna Bihari	Udaipur	Rajasthan
Silver Hospital	Surat	Gujarat
Satguru Clinic	Surat	Gujarat
Amar Health Care	Mathura	Uttar Pradesh
Abhishek Biswas	Kolkata	West Bengal
Surendra Kumar Sharma	Jaipur	Rajasthan
Satyaki Bhardhan	Kolkata	West Bengal
Nidaan Hospital	Gwalior	Madhya Pradesh
Dr Vk Agarwal Clinic	Agra	Uttar Pradesh
Harmony Health Hub	Nashik	Maharashtra
Essani Hospital	Hyderabad	Telangana
Surabhi Hospital	Karimnagar	Telangana
Kiran Diabetes Care	Hyderabad	Telangana
Deepu Balakrishnan	Trivandrum	Kerala
Janaraksha Hospitral	Coimbatore	Tamil Nadu
Madhan Mohan Clinic	Chennai	Tamil Nadu
Soumya Clinic	Thanjavur	Tamil Nadu
Mmm Hospital	Chennai	Tamil Nadu
Dr Shantanu Agarwal Clinic	Meerut	Uttarakhand
Maini Clinic	Mohali	Punjab
Gurpal Singh	Mohali	Punjab
Narayan Diabetese Clinic	Lucknow	Uttar Pradesh
Partha Sarathi Das	Siliguri	West Bengal
Jamirul Islam	Siliguri	West Bengal
Aanand Dialysis Centre	Jalgaon	Maharashtra
Dev Cardio , Sidhari	Varanasi	Uttar Pradesh
Dr Kamla Dalmia	Cuttack	Odisha
Snr Heart Center	Cuttack	Odisha
Dr Nirajan Mohaptra	Cuttack	Odisha
Sukh Sadan Clinic	Kangra	Himachal Pradesh
Lalit Shrimali	Udaipur	Rajasthan
Kumar Pramod	Muzaffarpur	Bihar
Priyadarshi Sailesh	Siliguri	West Bengal
Dot Clinic	Amritsar	Punjab
Metrocity Hospital	Meerut	Uttar Pradesh
Sghs Hospital	Mohali	Punjab
Lallan Prasad	Dibrugarh	Manipur
Anakal Endocrine Hospital	Gulbarga	Karnataka
Geetanjali Hospital	Udaipur	Rajasthan
Bt Medical	Guwahati	Assam
Naruvi Hospital	Chennai	Tamil Nadu
Fortis Hospital	Mohali	Punjab
Sidhi Vinayak Hospital	Ahmedabad	Gujarat
Suresh Kumar Bhawasinka	Muzaffarpur	Bihar
Abdul Hamid	Dibrugarh	Assam
Papular Hospital	Varanasi	Uttar Pradesh
Arora Heart Center	Meerut	Uttar Pradesh
Max Hospital Dehradun	Meerut	Uttar Pradesh
Dhanvai And Singh Hospital	Aurangabad	Maharashtra
Jawalekar Hospital	Nanded	Maharashtra
Patel Hospital	Latur	Maharashtra
